# Diversity of hemodynamic types in connective tissue disease associated pulmonary hypertension: more than a subgroup of pulmonary arterial hypertension

**DOI:** 10.1186/s12890-022-02081-0

**Published:** 2022-08-01

**Authors:** Xingbei Dong, Yue Shi, Ying Xia, Xiao Zhang, Junyan Qian, Jiuliang Zhao, Jinmin Peng, Qian Wang, Li Weng, Mengtao Li, Bin Du, Xiaofeng Zeng

**Affiliations:** 1 Department of Rheumatology and Clinical Immunology, Chinese Academy of Medical Sciences & Peking Union Medical College, National Clinical Research Center for Dermatologic and Immunologic Diseases (NCRC-DID), Ministry of Science & Technology, State Key Laboratory of Complex Severe and Rare Diseases, Peking Union Medical College Hospital (PUMCH), Key Laboratory of Rheumatology and Clinical Immunology, Ministry of Education, Beijing, 100730 China; 2 Medical Intensive Care Unit, Peking Union Medical College Hospital (PUMCH), Chinese Academy of Medical Sciences & Peking Union Medical College, Beijing, 100730 China

**Keywords:** Connective tissue disease, Pulmonary hypertension, Hemodynamics, PAWP, PVR, Prognosis

## Abstract

**Objective:**

Connective tissue disease associated pulmonary hypertension (CTD-PH) is classified as a subgroup of WHO group 1 PH, also called pulmonary arterial hypertension (PAH). However, not all CTD-PH fit hemodynamic definition of PAH. This study investigates the diversity of hemodynamic types of CTD-PH, their differences in clinical characteristics and outcomes.

**Method:**

We performed a retrospective cohort study. CTD-PH patients were enrolled and divided into WHO group1 PH, WHO group 2 PH and hyperdynamic PH (mPAP > 20 mmHg, PVR < 3WU, PAWP < 15 mmHg) according to hemodynamics obtained by right heart catheterization. Patients with severe lung diseases, heart failure with reduced ejection fraction, pulmonary embolism, and hepatic cirrhosis were excluded. Baseline characteristics, autoantibodies, cardiac function, echocardiogram parameters, hemodynamics and survival rates were compared.

**Result:**

A total of 202 CTD-PH patients were included, 138 in WHO group 1 PH, 33 in WHO group 2 PH and 31 in hyperdynamic PH. We found hyperdynamic PH is less severe, presenting lower NT-proBNP level, better WHO function class, lower mPAP and PVR, higher cardiac output, and less cardiac remodeling. Incidence of anti-RNP was significantly lower in patients with elevated PAWP. Short-term survival was worse in WHO group 2 PH, yet 5-year survival rates didn’t differ between groups.

**Conclusion:**

Considering diversity in hemodynamic types, CTD-PH is more than a subgroup of PAH. Different types of CTD-PH present different clinical phenotypes and outcome. Phenotyping PH in CTD-PH patients is important.

**Supplementary Information:**

The online version contains supplementary material available at 10.1186/s12890-022-02081-0.

## Introduction

Pulmonary hypertension (PH) is a pathophysiological disorder, that blood pressure in the pulmonary artery is elevated due to a variety of clinical conditions [1]. In the 6th World Symposium on Pulmonary Hypertension, PH is redefined as mean pulmonary arterial pressure (mPAP) > 20 mmHg at rest as assessed by right heart catheterization (RHC) [2]. PH is categorized into 5 groups according to different clinical aspects [2, 3]. WHO (World health organization) group 1 PH, also called pulmonary arterial hypertension (PAH), occurs as a consequence of pulmonary arterial morbidities; WHO group 2 PH is due to impaired left heart function and subsequent pulmonary vascular congestion; WHO group 3 PH is a result of chronic lung disease and/or hypoxia; PH caused by obstruction of the pulmonary artery is classified as group 4; And PH with unclear or multifactorial mechanisms is group 5.

PH is also a severe and frequent complication of various types of connective tissue diseases (CTD). The prevalence of PH in CTD is around 3–13%, and is proved to be associated with worse prognosis [4]. CTD associated PH are mostly results of isolated pulmonary vascular diseases, which affects pre-capillary arterioles. Therefore, it is categorized as a subgroup of PAH.

Both CTD and PH are heterogeneous diseases, thus CTD-PH has some unique clinical characteristic compared with other forms of PH. CTD is a group of systemic diseases, most of which can involve not only pulmonary arteries, but also pulmonary veins, myocardium, liver, lungs, or associate with venous thromboembolism, presenting with various clinical characteristics. Several mechanisms can work together and lead to PH, including pulmonary vasculopathy, pulmonary venous-occlusive disease, myocardium involvement, interstitial lung disease (ILD), pulmonary embolism (PE) and hepatic cirrhosis. Former large registries, reviews and case report have described PH other than PAH can be found in CTD patients, and the possible overlap of other forms of PH are numerous [5–8]. Furthermore, different treatment strategies are indicated for the different subgroups of PH [3]. In order to apply the most appropriate therapeutic option, carefully phenotyping PH in CTD patients become very important.

In clinical practice, a diagnosis of PAH is usually made after other causes of PH are excluded, such as left heart disease, sever lung disease, PE, and hepatic cirrhosis. However, following this diagnostic strategy, not all CTD-PH patients match hemodynamic definition of PAH [9]. Some may present with elevated pulmonary arterial wedge pressure (PAWP), and some show pulmonary vascular resistance (PVR) lower than 3WU with preserved or elevated cardiac output (CO) [10]. Meanwhile, a combination of ILD is also common in CTD-PAH patients, which often complicates the situation. Up till now, only a few studies have described characteristics and prognosis of CTD-PH that has elevated PAWP [11, 12] or that associated with ILD [13–15], few on CTD-PH with normal PVR [10]. More importantly, no study described the difference in disease phenotypes and outcomes of non-PAH portion of CTD-PH patients. Thus, we undertook this study to explore the diversity of hemodynamic phenotypes of CTD-PH, their difference in clinical characteristics and prognosis.

## Materials and methods

### Study population

This is a single center retrospective cohort study. All patients meet the wildly accepted criteria of CTD [16–22]. Patients who show risk factors for PH or symptoms related to PH were referred to transthoracic echocardiogram (TTE). Those with a peak tricuspid regurgitation velocity higher than 2.8 m/s or abnormalities of right atrial or right ventricle detected by TTE were suspected of PH, and underwent detailed workup and RHC. We enrolled patients who received RHC in medical intensive care unit (MICU) in our center from June 1, 2016 to February 1, 2020. All patients had at least 12 months of follow up. Patients with mPAP ≤ 20 mmHg by RHC, or associated with heart failure with reduced ejection fraction (HFrEF), congenital heart disease, severe lung disease, pulmonary embolism or hepatic cirrhosis were excluded from further comparative analysis. Severe lung disease was defined as FEV1 < 60% or FVC < 70% or signs of extensive parenchymal changes in high-resolution CT (HRCT) of the lungs [6, 23].

### Group definition

Patients were divided into three groups according to their RHC results. The patients is WHO group 1 PH if mPAP > 20 mmHg, PAWP ≤ 15 mmHg, and PVR > 3WU. The patient is WHO group 2 PH if mPAP > 20 mmHg while PAWP > 15 mmHg. The other patients have mPAP > 20 mmHg, PAWP ≤ 15 mmHg while PVR < 3WU, which cannot be categorized into any hemodynamic types of PH. Thus, we defineed these patients as “hyperdynamic PH”. WHO group 2 PH were further divided into isolated post-capillary PH (IpcPH, PVR < 3WU) and combined pre-capillary and post-capillary PH (CpcPH, PVR ≥ 3WU) for subgroup analysis.

### Data collection

The following variables were selected as clinically important: age, sex, specific diagnosis of CTD, disease duration till PH onset, presence of co-morbidities (including hypertension, diabetes, heart diseases and thyroid disease), inflammatory markers, autoantibodies, WHO function class, B-type natriuretic peptide (BNP) and N-terminal pro-BNP (NT-proBNP) levels, hemodynamic parameters measure by RHC and echocardiographic findings. All data were collected within 2 weeks before performance of RHC. Baseline characteristic, disease characteristics, 1-year and 5-years survival rates are compared between the 3 groups.

### Statistical analysis

Mean and standard deviations were used to describe parametric data. Grouped-t test, Anova analysis, and Kruskal–Wallis H test were used to compare continuous variables. The Kruskal–Wallis H test was used for non-normal distributed variables. X^2^ or Fisher exact test to compare categorical variables. *p* value < 0.05 was considered statistically significant. Survival analysis was performed with Kaplan–Meier analysis. Baseline for survival analysis is the date of RHC, and death due to all cause was defined as terminal incident. SPSS, version 21 statistical software was used for all analysis (Additional file 1).

## Results

### Hemodynamic phenotypes and clinical phenotypes of CTD-PH

A total of 236 CTD patients were suspected of PH screened by TTE. Among them, 3 patients were excluded because of HFrEF, 4 were exclude because of congenital heart disease, 9 were excluded because of severe lung disease, 2 were excluded for signs of PE on CTPA, and 3 were excluded because of pulmonary arteritis. Among patients with severe lung disease, 7 were restrictive lung disease, 1 was obstructive lung disease, and 1 show diffused interstitial change on HRCT with decreased diffusing capacity (DLCO). A total of 215 patients were suspected of PH and underwent RHC in Medical intensive care unit in Peking Union Medical College Hospital. Another 13 patients were excluded from the study because of normal mPAP (≤ 20 mmHg), leading to a cohort of 202 CTD-PH patients.

**Fig. 1:**
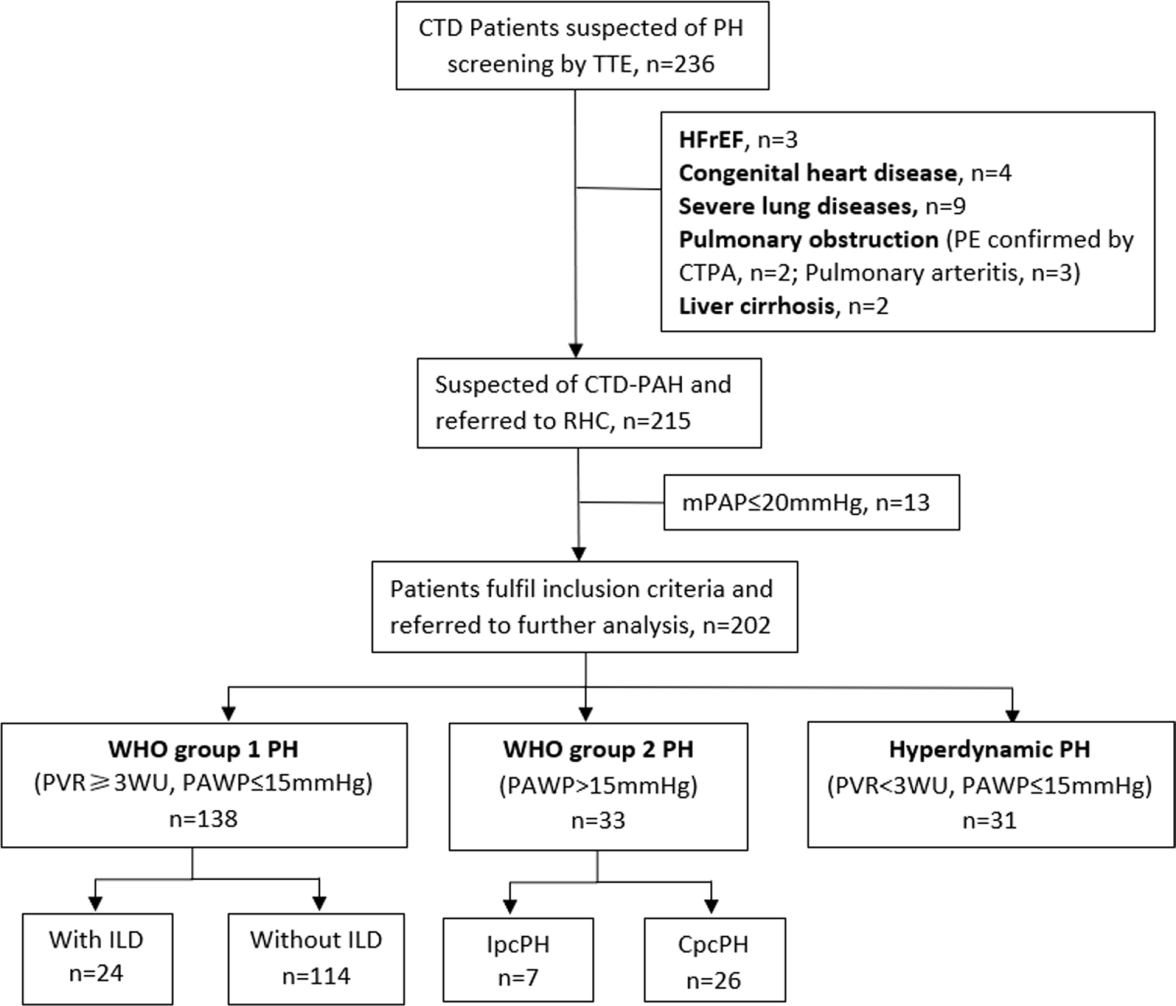
Flow chart of patient selection. 34 patients were excluded (Patients with mPAP < 20 mmHg, n = 13; Patients with HFrEF, n = 3; Patients with congenital heart disease, n = 4; Patients with severe lung disease, n = 9; Patients with pulmonary embolism, n = 2; Patients with pulmonary arteritis, n = 3; Patients with live cirrhosis, n = 2). *CTD* Connective tissue disease, *PH* Pulmonary hypertension, *TTE* Transthoracic echocardiography, *HFrEF* Heart failure with duce ejection fraction, *PE* Pulmonary embolism, *CTPA* Computed tomography pulmonary angiogram, *PAH* Pulmonary arterial hypertension, RHC Right heart catheterization, *mPAP* Mean pulmonary artery pressure, *WHO* World Health Organization, *PAWP* Pulmonary arterial wedge pressure, *PVR* Pulmonary vascular resistance, *ILD* Interstitial lung disease, *IpcPH* Isolated post-capillary pulmonary hypertension, *CpcPH* Combined pre-capillary and post-capillary pulmonary hypertension

Finally, a study cohort of 202 patients were included and divided into 3 groups. According to hemodynamic parameter, 33 patients were in WHO group2 PH, 31 patients were hyperdynamic PH and 138 were WHO group 1 PH. (See Fig. 1).

**Fig. 2:**
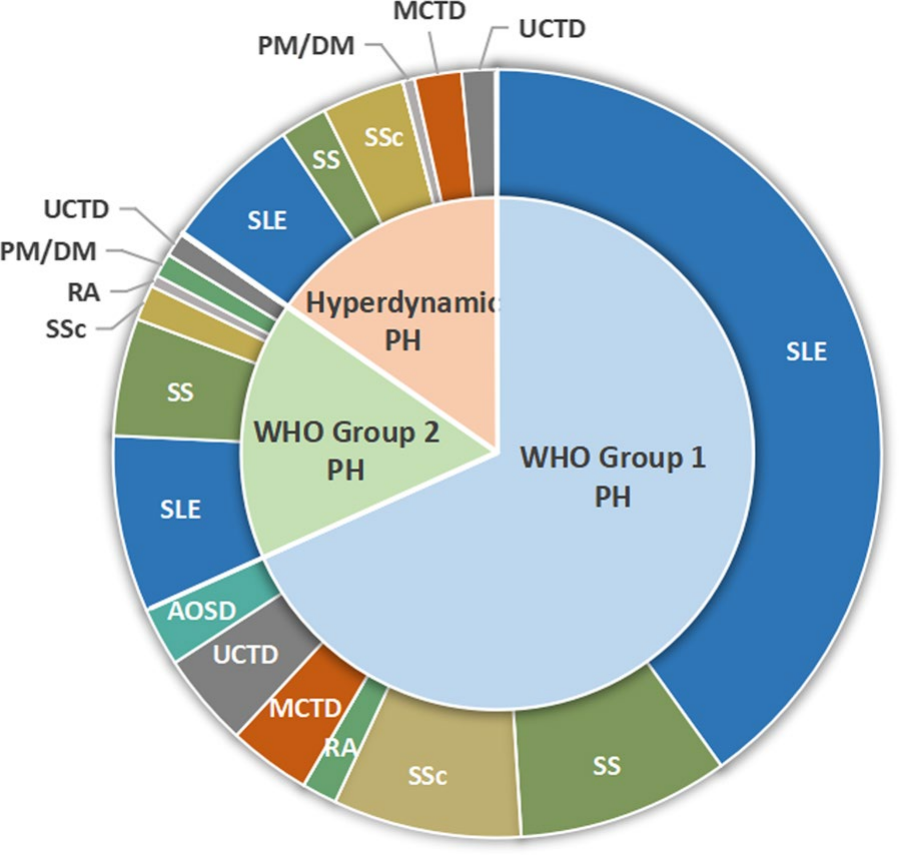
Spectrum of different types of CTDs in CTD-PH patients. *WHO* World health organization, *PH* Pulmonary hypertension, *SLE* Systemic lupus erythematosus, *SS* Sjogren syndrome, *SSc* Systemic sclerosis, *RA* Rheumatoid arthritis, *PM* Polymyositis, *DM* Dermatomyositis, *MCTD* Mixed connective tissue disease, *UCTD* Undifferentiated connective tissue disease, *AOSD* Adult-onset Still’s disease

Among all 203 patients included, systemic lupus erythematosus (SLE) was the most common CTD associates with PH, with a prevalence of 53.5% (n = 108). Sjogren syndrome (SS)-PH and systemic sclerosis (SSc)-PH also took a high percentage in this cohort, the prevalence was 15.8% (n = 32) and 12.9% (n = 26) respectively. Our study also included small groups of patients with rheumatoid arthritis (RA)-PH (n = 4, 2.0%), polymyositis or dermatomyositis (PM/DM)-PH (n-3, 1.5%), mixed connective tissue disease (MCTD)-PH (n = 11, 5.4%), undifferentiated connective tissue disease (UCTD)-PH (n = 13, 6.4%), adult-onset Still’s disease (AOSD)-PH (n = 5, 2.5%). The spectrum of CTD in the 3 groups didn’t show significant difference (*p* > 0.05) (See Fig. 2 and Table 1).

**Table 1:** Demographic features of patients with WHO group 1 PH, WHO group 2 PH, and hyperdynamic PH

	WHO group 1 PH n = 138	WHO group 2 PH n = 33	Hyperdynamic PH n = 31	*p* value
Age, years	36.6 ± 11.6	39.9 ± 12.7	36.6 ± 11.6	0.239
Female, No. (%)	135 (97.8)	32 (97)	31 (100)	0.198
BMI, kg/m^2^	21.3 ± 3.1	22.1 ± 5.1	22.6 ± 2.9	0.785
Disease duration since onset of CTD, weeks	41.8 ± 60.6	23.1 ± 48.0	35.0 ± 49.4	**0.039**
Dyspnea on exertion, No. (%)	116 (84.1)	27 (81.8)	22 (71.0)	0.235
Interstitial lung disease, No. (%)	24 (17.4)	3 (9.1)	9 (29.0)	0.112
Hypertension, No. (%)	14 (10.1)	0 (0)	4 (12.9)	0.090
Diabetes, No. (%)	3 (2.2)	1 (3.0)	1 (3.2)	0.653
Hyperthyroid, No. (%)	2 (1.4)	1 (3.0)	0 (0)	0.683
Hypothyroid, No. (%)	13 (9.4)	1 (3.0)	4 (12.9)	0.333
Pregnancy, No. (%)	0 (0)	0 (0)	0 (0)	1.000
Anemia (HGB < 90 g/L), No. (%)	2 (1.5)	3 (9.1)	1 (3.2)	0.056
Diagnosis of CTD, No. (%)				
SSc	16 (11.6)	3 (9.1)	7 (22.6)	0.259
Other CTDs				
SLE	81 (58.7)	15 (45.5)	12 (38.7)	
SS	18 (13.0)	10 (30.3)	4 (12.9)	
RA	3 (2.2)	1 (3.0)	0 (0)	
PM/DM	0 (0)	2 (6.1)	1 (3.1)	
MCTD	7 (5.1)	0 (0)	4 (12.9)	
UCTD	8 (5.8)	2 (6.1)	3 (9.7)	
AOSD	5 (3.6)	0 (0)	0 (0)	

*p*-values <0.05 is shown in bold, which means statistically significant

*WHO* World Health Organization, *PH* Pulmonary hypertension, *BMI* Body mass index, *CTD* Connective tissue disease, *HGB* Hemoglobulin, *SSc* Systemic sclerosis, *SLE* Systemic lupus erythematosus, *SS* Sjogren syndrome, *RA* Rheumatoid arthritis, *PM* Polymyositis, *DM* Dermatomyositis, *MCTD* Mixed connective tissue disease, *UCTD* Undifferentiated connective tissue disease, *AOSD* Adult-onset Still’s disease

### Demographic features, disease characteristics and treatment regimen between WHO group 1 PH, WHO group 2 PH and hyperdynamic PH

CTD-PH patients in our cohort were young (37.0 ± 11.9 years old), predominantly female (n = 199, 98.5%), and had a mean BMI of 21.7 ± 3.5 kg/m^2^. The mean duration between onset of CTD and diagnosis of PAH was 37.8 ± 57.3 months. Patients with WHO group 2 PH tend to have longer disease duration since onset of CTD to development of PH. Other baseline characteristics did not differ significantly between 3 groups (Table 1).

Laboratory findings, including immunoglobulin G, compliment level, erythrocyte sedimentation rate, hypersensitive C-reactive protein and autoantibodies were compared between the three groups. Incidence of anti-ribonucleoprotein (anti-RNP) was significantly higher in WHO group 1 PH and hyperdynamic PH (Table 2).

**Table 2:** Disease characteristics of WHO group 1 PH, WHO group 2 PH, and hyperdynamic PH

	WHO group 1 PH n = 138	WHO group 2 PH n = 33	Hyperdynamic PH n = 31	*p* value
IgG (g/L)	17.1 ± 8.4	18.8 ± 10.9	17.7 ± 7.4	0.735
Hypocomplementemia, No. (%)	40 (31.7)	12 (42.9)	9 (29.0)	0.454
Elevated hsCRP or ESR, No. (%)	70 (56.0)	15 (60.0)	14 (50.0)	0.773
Autoantibodies, No. (%)				
ANA	130 (97.7)	30 (100.0)	30 (96.8)	0.782
Anti-dsDNA	35 (25.4)	5 (15.2)	7 (22.6)	0.268
Anti-Sm	25 (18.1)	1 (3.0)	5 (16.1)	0.147
Anti-RNP	76 (55.1)	11 (33.3)	24 (77.4)	**0.005**
Anti-SSA	76 (55.1)	22 (66.7)	17 (54.8)	0.414
Anti-SSB	22 (15.9)	10 (30.3)	5 (16.1)	0.161
Anti-Scl-70	1 (0.7)	0 (0)	2 (6.5)	0.186
Anti-Ro-52	72 (52.2)	17 (51.5)	12 (38.7)	0.554
Anti-β2GP1	10 (7.2)	1 (3.0)	3 (9.7)	0.391
ACL	3 (2.2)	2 (6.1)	1 (3.2)	0.505
Elevated LA	3 (2.2)	1 (3.0)	2 (6.5)	0.181
BNP, ng/L	137.0 ± 275.0	221.4 ± 322.5	36.2 ± 45.8	**0.047**
NT-proBNP, pg/ml	1236.2 ± 2777.8	1290.0 ± 1882.2	112.4 ± 118.4	** < 0.001**
WHO function class III–IV, No. (%)	23 (16.7)	5 (15.2)	0 (0)	**0.028**
Hemodynamics				
mABP, mmHg	90 ± 10	90 ± 13	92 ± 9	0.447
mPAP, mmHg	45 ± 11	49 ± 11	25 ± 4	< **0.001**
PAWP, mmHg	10 ± 3	19 ± 3	12 ± 2	< **0.001**
RAP, mmHg	7 ± 3	12 ± 4	7 ± 2	< **0.001**
CO, L/min	5.2 ± 1.6	5.3 ± 1.6	6.8 ± 1.3	< **0.001**
CI, L/min × m^2^	3.3 ± 0.8	3.4 ± 1.1	4.2 ± 0.8	< **0.001**
PVR, WU	7.5 ± 3.8	6.7 ± 4.4	1.9 ± 0.6	< **0.001**
Echocardiography				
IVC diameter, mm	14.2 ± 3.0	14.6 ± 2.0	13.0 ± 2.0	**0.041**
RV diameter, mm	27.7 ± 7.2	27.9 ± 6.0	21.8 ± 4.4	< **0.001**
RV/LVEDD ratio	0.70 ± 0.27	0.64 ± 0.16	0.47 ± 0.10	< **0.001**
LVEF %	69.2 ± 6.2	65.7 ± 8.6	67.0 ± 7.6	0.110
TAPSE, mm	17.2 ± 3.5	18.1 ± 3.6	19.0 ± 3.7	0.456
Mitral valve regurgitation, No. (%)	14 (13.0)	9 (40.9)	5 (17.9)	**0.028**
Pericardial effusion, No. (%)	36 (33.6)	10 (34.5)	4 (13.8)	0.104

*p*-values <0.05 are shown in bold, which means statistically significant

*WHO* World Health Organization, *PH* Pulmonary hypertension, *IgG* Immunoglobulin G, *hsCRP* Hypersensitive C-reaction protein, *ESR* Erythrocyte dissemination rate, *ANA* Anti-nuclear antibodies, *anti-dsDNA* anti-double-stranded DNA, *anti-Sm* Anti-Smith, *ACL* Anticardiolipin, *Anti-β2GP1* Anti-beta2 glycoprotein 1, *anti‐RNP* Antiribonucleoprotein, *BNP* Brain natriuretic peptide, *NT-proBNP* N-terminal brain natriuretic peptide, *mABP* Mean arterial blood pressure, *mPAP* Mean pulmonary pressure, *PAWP* Pulmonary arterial wedge pressure, *RAP* Right atrium pressure, *CO* Cardiac output, *CI* Cardiac index, *PVR* Pulmonary arterial resistance, *IVC* Inferior vena cava, *RV* Right ventricle, *LVEDD* Left ventricular end-diastolic diameter, *LVEF* Left ventricular ejection fraction, *TAPSE* Tricuspid annular plane systolic excursion

As for characteristic of PH, patients with hyperdynamic PH were less severe than the other two groups, with lower NT-proBNP level, better WHO function class, lower mean pulmonary artery pressure, and much higher cardiac output. Hyperdynamic PH also has smaller right ventricular diameter on TTE, indicating less ventricular remodeling. Patients with WHO group 2 PH were more likely to have mitral valve regurgitation, and higher right atrium pressure (RAP) than the other 2 groups (Tables 1, 2).

Some patients in our cohort had PAH targeted drugs before RHC. To eliminate the influence of PAH-targeted drugs on hemodynamics, we also performed a subgroup analysis of treatment-naïve patients. The results were similar with the whole cohort (Additional file 2: Tables S1, S2).

In our cohort, the proportion of patients that received PAH-targeted therapy after RHC was 83.3%, 60.6% and 35.5% respectively in WHO group 1 PH, WHO group 2 PH and hyperdynamic PH. 29% of hyperdynamic PH patients discontinued PAH-targeted drugs after RHC, because they no longer satisfy the criteria of PAH. 22 patients were previously treated with PAH-targeted medication and later-on found out to be WHO group2 PH. All patients with IpcPH (n = 4) discontinued the PAH-targeted medication after RHC, while 20 CpcPH maintained or add PAH-targeted drugs (Table 3 and Additional file 2: Table S3).

**Table 3:** PAH-targeted therapy before and after RHC in WHO group 1 PH, WHO group 2 PH, and hyperdynamic PH

	WHO group 1 PH n = 138	WHO group 2 PH n = 33	Hyperdynamic PH n = 31	*p* value
PAH-targeted therapy before RHC, No. (%)	70 (50.7)	22 (66.7)	20 (64.5)	0.151
PAH-targeted therapy after RHC, No. (%)	103 (83.3)	20 (60.6)	11 (35.5)	< **0.01**
Monotherapy, No. (%)	89 (64.5)	13 (39.4)	8 (25.8)	
Combined therapy, No. (%)	25 (18.1)	7 (21.2)	3 (9.7)	
ERA, No. (%)	60 (43.8)	12 (37.5)	7 (22.6)	
PDE-I, No. (%)	79 (57.7)	14 (43.8)	7 (22.6)	
PGs, No. (%)	3 (2.2)	0 (0)	0 (0)	
GCA, No. (%)	0 (0)	0 (0)	1 (3.2)	

*p*-values <0.05 is shown in bold, which means statistically significant

*PAH* Pulmonary arterial hypertension, *RHC* Right heart catheterization, *WHO* World Health Organization, *PH* Pulmonary hypertension, *ERA* Endothelin receptor antagonist, *PDE-I* Phosphodiesterase inhibitor, *PGs* Prostaglandin analogs, *GCA* Guanylate cyclase agonist

### IpcPH and CpcPH in WHO group 2 PH patients

Of the 33 patients with PAWP > 15 mmHg, 26 (78.8%) were classified as CpcPH and 7 (21.2%) were classified as IpcPH based on whether their PVR is higher than 3 Wood Unit [2]. Though not statically different, CpcPH patients tend to have higher BNP and NT-proBNP level, higher mPAP and larger right ventricle diameter (Additional file 2: Table S3).

### CTD-PAH with ILD and without ILD

Among the 138 WHO group 1 PH patients, 24 (17.4%) were confirmed by HRCT and lung function test to be associated with mild to moderate ILD. Except for patients with ILD were older than patients without ILD, other epidemiology characteristics were similar between two groups. No significant differences were found in hemodynamics nor cardiac parameters (Additional file 2: Table S4).

### Survival between WHO group1 PH, WHO group 2 PH and hyperdynamic PH

**Fig. 3:**
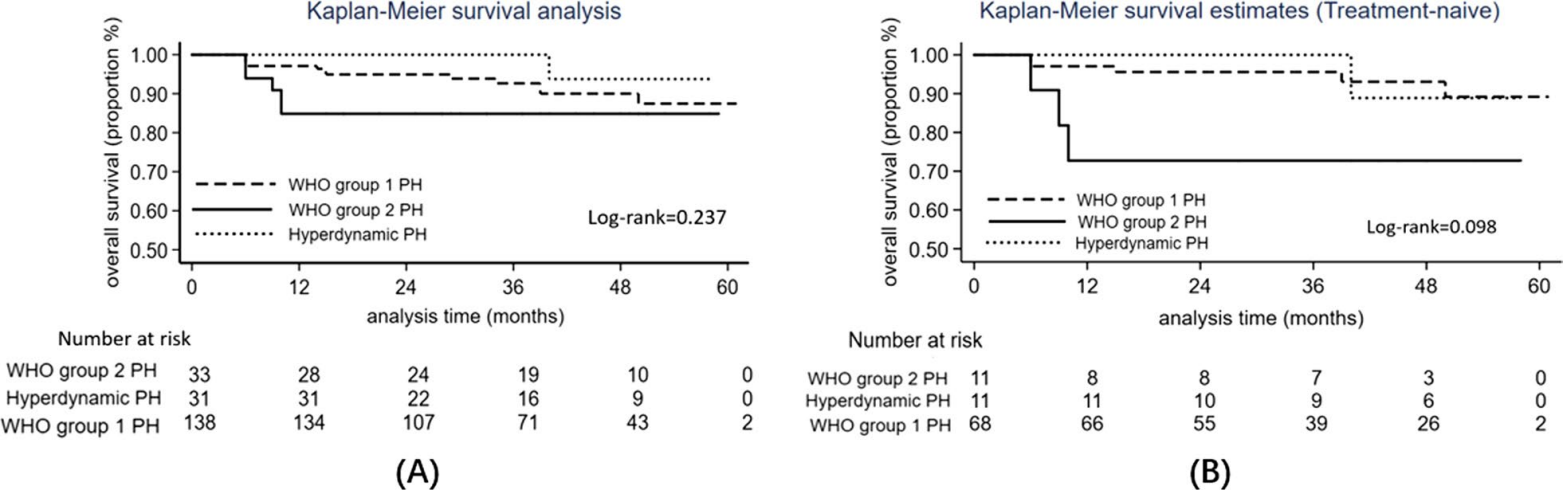
Kaplan–Meier curves for all-cause mortality of WHO group 1 PH, WHO group 2 PH, and Hyperdynamic PH. **A** Kaplan–Meier survival analysis between 3 group in the whole cohort. 5-year survival rate showed no significant difference (*p* = 0.237); **B** Kaplan–Meier survival analysis between 3 groups in treatment-naïve patients. 5-year survival rate showed no significant difference (*p* = 0.098)

The survival analysis has been performed between three groups (Fig. 3). Over a 5-year observation period, a total of 19 patients died, leading to a survival rate of 90.6%. Short-term survival was significantly different between three groups. One-year survival rates of WHO group 1, Group 2 and hyperdynamic PH was 97.1%, 84.8% and 100% respectively (*p* = 0.004). However, 5-year survival rate showed no significant difference (87.5%, 84.8%, versus 93.8%, *p* = 0.237). There were no differences detected in survival between IpcPH and CpcPH (85.7% versus 84.6%, *p* = 0.984). Co-existence of ILD was not a risk factor for death before (HR = 0.563, 95%CI 1.30–2.45, *p* = 0.731) or after adjusted for age (HR = 0.21, 95%CI 0.04–1.02).

## Discussion

To our knowledge, this is the first study comparing clinical characteristic between different hemodynamic phenotypes of CTD-PH, and the first study to describe the characteristic of a group of CTD-PH patients with PVR < 3WU and elevated CO (hyperdynamic PH), which was less severe considering hemodynamic and cardiac parameters, but with similar 5-year outcome compared with CTD-PAH.

CTD is a type of disease that affect multiple organs and systems. PH is one of the common complications of CTD, and can hamper prognosis. Although CTD-PH is currently a subcategory of WHO group 1 PH, also known as PAH. According to our cohort, PAH is not the only hemodynamic phenotype of CTD-PH. Among the 218 patients who confirmed PH by RHC, 4.1% (9 cases) were PH caused by lung disease or hypoxia after assessment of lung function test and chest high-resolution CT, 0.9% (2 cases) were CTEPH according to CT pulmonary angiography, and finally 16.5% (36 cases) were PH caused by LHD with PAWP > 15 mmHg confirmed by RHC. Since our hospital is a rheumatic disease referring center, it is highly likely that the non-PAH proportion, which caused by complications mentioned above, may be underestimated.

SSc-PH accounts for approximately 50–70% of CTD-PH in western large registries [4, 6], whereas PH associated with SLE is more common in China [24]. SLE, SS and SSc took up most of the CTD-PH patients in our cohort, prevalence of which are 49.8%, 16.4% and 13.7%. PH develops much more uncommonly with RA, vasculitis, PM or DM [25, 26], and is rarely seen in patients with AOSD, only a few cases have been reported [27]. However, because of lacking large cohorts, prevalence of PH in patients with CTDs other than SSc remains to be further determined. In our cohort, 5 patients with AOSD, 4 patients with RA, 4 patients with systemic vasculitis and 3 patients with PM/DM have been included. Regarding the aspect of PH, these cases include not only PAH, but also PH due to left heart disease and lung disease. The exact pathophysiology of how PH develops in RA, PM/DM, AOSD or systemic vasculitis is yet unknown. As has been hypothesized with other CTDs, endothelial dysfunction and remodeling of pulmonary arteries, cardiac involvement, ILD, PE as well as immune dysregulation can all play a role.

Recently, attention has been driven to a group of patients with clearly elevated mPAP (≥ 25 mmHg) and without relevant LHD (PAWP ≤ 15 mmHg), who fail to fulfill the hemodynamic criteria of pre-capillary PH because of “normal” PVR (PVR ≤ 3 WU). A few studies have suggested that PVR ≥ 2WU is already associated with PH [10, 28]. Data of an article published by Xanthouli et al. [10] showed that patients with PVR ≥ 2WU who still have a preserved CO at rest (5.47 ± 1.11L/min, 95% CI 5.04–5.90L/min), have already presented with impaired exercise capacity, right heart function and worse prognosis. The result of our study also addresses this point. All 31 patients in hyperdynamic PH group exhibited low PVR (1.9 ± 0.6WU, 95%CI 1.7–2.2) and preserved CO (6.8 ± 1.3L/min, 95% CI 6.3–7.3L/min). No patients had co-existing conditions that can cause hyperdynamic circulatory state, such as pregnancy, hyperthyroid, severely anemia, or hepatic cirrhosis. Former studies focusing on right ventricle demonstrated that the right ventricle is very sensitive to afterload changes and its adaptation to chronic afterload involves increasing contractility [29]. Thus we hypothesized that PVR ≥ 2WU has already caused an increase in pulmonary circulation afterload, and the cardiac function is preserved or correspondingly increased at this time to compensate. When the cut off value for PVR is set too high (3WU), the elevated CO will be regarded as a "hyperdynamic" state.

The contribution of LHD to PH in CTD patients is not yet well established. Current data mostly comes from SSc-PH. Cardiac involvement is common in SSc. Studies have reported that myocardial fibrosis is the pathological hallmark of this complication, and has been proved by cardiac MRI as well as biopsies [5]. This can either explain the development of post-capillary PH or add post-capillary component to pre-capillary PH (CpcPH). Other CTDs, such as SLE, RA, PM/DM are also known to involve cardiac muscles, and can subsequently be associated with WHO group 2 PH. In our study, most patients classified as WHO group 2 PH have preserved ejection fraction (LVEF 68.2 ± 7.0%, 2 patients had LVEF between 40–45%), which is consistent with prior studies [11, 12]. Furthermore, we discovered that WHO group 2 PH is more likely to associate with mitral valve dysfunction than WHO group 1 PH. Although because of the low prevalence, valvular involvement is not considered a typical manifestation of SSc or SLE [30], but it could contribute to the development of PH in CTD patients. CpcPH demonstrated higher mPAP and right ventricle diameter than IpcPH, indicating a worse hemodynamic and structural state. Bourji KI et al. reported CpcPH demonstrates worse survival [12]. However, Lammi MR et al. [11] reported that survival was similar between IpcPH and CpcPH. Our study also failed to find difference in survival between these two groups. But more data is required to support this conclusion.

ILD is a frequent complication of CTD which can be detected by high-resolution CT and lung function test. Morrisroe et al. [13] found that ILD is an independent predictor of death in CTD-PH patients, and assumed that co-existence of ILD could lead to a more severe clinical phenotype. However, results from another study showed no association between the severity of ILD and hemodynamic profiles [14]. Michelfelder et al. [15] compared SSc-PAH-ILD (n = 24) patients with SSc-PAH patients (n = 27), and did not find a difference in hemodynamic parameters, NT-proBNP levels, FVC/DLCO ratio, 6 MW, WHO function class and scleroderma-specific autoantibody levels between two groups, but a decreased survival rate in SSc-PAH-ILD patients. Consistent with former studies, our study found hemodynamics, echocardiogram parameters were of no significant difference regardless of the association with ILD. However, t is still under debate whether co-existing of ILD increases the risk of death in CTD-PH patients. And it seems HRCT, lung function test and RHC are not reliably enough to distinguish between WHO group 1 and group 3 PH. Further studies are needed to answer these questions.

Our study also addresses the importance of carefully phenotyping PH in CTD-PH patients in order to provide the most appropriate treatment. 4 out of 7 CTD-PH patients with post-capillary PH were on PAH targeted therapy before RHC, and discontinued the medication after. This is probably because primary hospitals lack the condition to perform RHC, and weren’t aware of the diversity of hemodynamic types of CTD-PH. Furthermore, hemodynamic classification may change over time. As shown in PHAROS cohort, 30% of SSc-PAH experienced PAWP class change during follow-up [31]. 76.9% of CpcPH patients in our cohort were treated with PAH-targeted therapy after RHC. Although some recent studies [32, 33] showed patients with CpcPH and HFpEF may benefit from phosphodiesterase type 5 inhibitor, evidence from randomized trial is still needed to determine whether PAH-targeted therapy can be applied in CpcPH. In any case, CpcPH patients should be monitored closely and regularly repeat RHC when taking PAH-specific drugs.

What is the best approach to distinguish the non-PAH proportion in CTD-PH patients? After ruling out patients with HFrEF, severe lung disease (FEV1 < 60% or FVC < 70% or signs of extensive parenchymal changes on HRCT of the lungs), and CTEPH (indication of pulmonary embolism on CTPA), still 64 patients (31.7%) cannot fit criteria for pre-capillary PH. Meanwhile, different forms of PH can overlap in one single patient and complicate the case. Unfortunately, our study failed to distinguish these patients in the perspective of CTD characteristics such as inflammatory markers and autoantibodies, except for anti-RNP positivity is lower in CTD associated with WHO group 2 PH. Thus, a full work up of echocardiography, lung function test, chest CT, CTPA or V/Q scan and most importantly RHC is still necessary when assessing CTD-PH patients.

Our study has limitations that must be acknowledged. Firstly, using only TTE as the only detection method for PH may miss patients with early PH [34]. Secondly, because this is a retrospective study, missing data were unavoidable. Thirdly, not all patients are included at the diagnostic RHC and some patients have already taken PH specific therapies, which could affect the results, since hemodynamic features can change during the course of disease. Lastly, the sample size of some subgroups is small and the risk for type 2 error exists.

## Conclusion

Our study showed that PAH is not the only hemodynamic type of CTD-PH, emphasizing the importance of carefully phenotyping of PH in CTD patients. We described a group of patients with PVR lower than 3WU and elevated cardiac output with better hemodynamic status and better short-term survival, which also is probably an indication that a PVR threshold of ≥ 3WU is too high to enable a diagnosis of PH. We also found CTD-PH with elevated PAWP has lower incidence of anti-RNP, and associate with worse short-term survival. However, a full workup including RHC is still needed to clearly distinguish non-PAH proportion of CTD-PH patients. Further investigations are still required to analyze the disease characteristic and prognostic difference of different hemodynamic subtypes of CTD-PH, and hopefully develop a better algorithm in assessing CTD-PH patients.

## Supplementary Information

**Additional file 1.** Raw dataset used and analysed during the current study.

**Additional file 2. Table S1.** Demographic features of patients with WHO group1 PH, WHO group2 PH, and hyperdynamic PH (treatment-naïve patients). **Table S2.** Disease characteristics of WHO group1 PH, WHO group2 PH, and hyperdynamic PH (treatment-naïve patients). **Table S3.** Baseline and disease characteristics for IpcPH and CpcPH. **Table S4.** Baseline and disease characteristics for CTD-PH patients with and without ILD.

## Data Availability

Raw dataset used and analysed during the current study was provided in additional file 1.

## Abbreviations

ANA: Anti-nuclear antibodies
anti-dsDNA: Anti-double-stranded DNA
anti-Sm: Anti-Smith
anti-β2GP1: Anti-beta2 glycoprotein 1
anti‐RNP: Antiribonucleoprotein
ACL: Anticardiolipin
AOSD: Adult-onset Still’s disease
BNP: B-type natriuretic peptide
BMI: Body mass index;
CO: Cardiac output
CI: Cardiac index
CTD: Connective tissue disease
CTPA: CT pulmonary angiography
CpcPH: Combined pre-capillary and post-capillary PH
DM: Dermatomyositis
ESR: Erythrocyte dissemination rate
GPA: Granulomatosis with polyangiitis
HGB: Hemoglobulin
HFrEF: Heart failure with reduced ejection fraction
HFpEF: Heart failure with preserved ejection fraction
HRCT: High-resolution computed tomography
hsCRP: Hypersensitive C-reaction protein
IgG: Immunoglobulin G
ILD: Interstitial lung disease
IpcPH: Isolated post-capillary PH
LHD: Left heart disease
LVEDD: Left ventricular end-diastolic diameter
LVEF: Left ventricular ejection fraction
IVC: Inferior vena cava
MCTD: Mixed connective tissue disease
mABP: Mean arterial blood pressure
mPAP: Mean pulmonary arterial pressure
NT-proBNP: N-terminal pro-BNP
RHC: Right heart catheterization
SLE: Systemic lupus erythematosus
SS: Sjogren syndrome
SSc: Systemic sclerosis
TTE: Transthoracic echocardiogram
PAH: Pulmonary arterial hypertension
PAWP: Pulmonary arterial wedge pressure
PE: Pulmonary embolism
PH: Pulmonary hypertension
PM: Polymyositis
PVR: Pulmonary vascular resistance
RA: Rheumatoid arthritis
RAP: Right atrium pressure
RV: Right ventricle
UCTD: Undifferentiated connective tissue disease
WU: Wood unit
WHO: World health organization

## Declarations

### Methods

All methods were carried out in accordance with relevant guidelines and regulations.
