# Supplementary material for: Diversity of hemodynamic types in connective tissue disease associated pulmonary hypertension: more than a subgroup of pulmonary arterial hypertension
Source: BMC Pulm Med. 2022 Aug 1;22:295. doi: 10.1186/s12890-022-02081-0 (PMC9341111; doi:10.1186/s12890-022-02081-0)
Supplement: Supplementary file 2 — Additional file 2. Table S1. Demographic features of patients with WHO group1 PH, WHO group2 PH, and hyperdynamic PH (treatment-naïve patients). Table S2. Disease characteristics of WHO group1 PH, WHO group2 PH, and hyperdynamic PH (treatment-naïve patients). Table S3. Baseline and disease characteristics for IpcPH and CpcPH. Table S4. Baseline and disease characteristics for CTD-PH patients with and without ILD. [file 12890_2022_2081_MOESM2_ESM.docx]

**Table S1—Demographic features of patients with WHO group1 PH, WHO group2 PH, and hyperdynamic PH（treatment-naïve patients）**

|  | **WHO group 1 PH**  **n=68** | **WHO group 2 PH**  **n=11** | **Hyperdynamic PH**  **n=11** | **p-value** |
| --- | --- | --- | --- | --- |
| **Age, years** | 36.7±12.2 | 42.5±16.5 | 46.0±13.6 | 0.066 |
| **Female, No. (%)** | 66 (97.8) | 11 (100) | 11 (100) | 1.000 |
| **BMI, kg/m2** | 21.2±2.6 | 22.9±6.1 | 24.4±3.0 | 0.052 |
| **Disease duration since onset of CTD, weeks** | 44.5±65.3 | 5.5±8.6 | 36.3±34.7 | 0.049 |
| **Dyspnea on exertion, No. (%)** | 61 (89.7) | 9 (81.8) | 5 (81.8) | 0.004 |
| **Interstitial lung disease, No. (%)** | 15 (22.1) | 1 (9.1) | 4 (36.4) | 0.323 |
| **Hypertension, No. (%)** | 10 (14.7) | 0 (0) | 1 (9.1) | 0.642 |
| **Diabetes, No. (%)** | 0 (0) | 1 (9.1) | 0 (0) | 0.362 |
| **Hyperthyroid, No. (%)** | 0 (0) | 0 (0) | 0 (0) | 1.000 |
| **Hypothyroid, No. (%)** | 6 (8.8) | 0 (0) | 1 (9.1) | 0.831 |
| **Pregnancy, No. (%)** | 0 (0) | 0 (0) | 0 (0) | 1.000 |
| **Moderate to severe anemia (HGB<90g/L), No. (%)** | 2 (2.9) | 1 (9.1) | 0 (0) | 0.573 |

WHO, World Health Organization; PH, pulmonary hypertension; BMI, Body mass index; CTD, connective tissue disease; HGB, hemoglobulin.

**Table S2—Disease characteristics of WHO group1 PH, WHO group2 PH, and hyperdynamic PH (treatment-naïve patients)**

|  | **WHO group 1 PH**  **n=68** | **WHO group 2 PH**  **n=11** | **Hyperdynamic PH**  **n=11** | **p-value** |
| --- | --- | --- | --- | --- |
| **IgG (g/L)** | 19.1±9.8 | 17.1±9.5 | 18.9±7.8 | 0.812 |
| **Hypocomplementemia, No. (%)** | 27 (44.3) | 4 (40) | 1 (9.1) | 0.091 |
| **Elevated hsCRP or ESR, No. (%)** | 41 (65.1) | 4 (44.4) | 4 (50) | 0.554 |
| **Autoantibodies, No. (%)** |  |  |  |  |
| **ANA** | 64 (94.1) | 9 (91.8) | 11 (100) | 1.000 |
| **Anti-dsDNA** | 18 (26.5) | 2 (18.2) | 2 (18.2) | 0.390 |
| **Anti-Sm** | 16 (23.5) | 1 (9.1) | 0 (0) | 0.065 |
| **Anti-RNP** | 45 (66.2) | 4 (36.4) | 8 (72.7) | **0.048** |
| **Anti-SSA** | 35 (51.5) | 7 (63.6) | 4 (36.4) | 0.198 |
| **Anti-SSB** | 9 (13.2) | 4 (36.4) | 0 (0) | **0.015** |
| **Anti-Scl-70** | 1 (1.5) | 0 (0) | 1 (1.5) | 0.193 |
| **Anti-Ro-52** | 35 (51.5) | 3 (27.3) | 4 (36.4) | 0.431 |
| **Anti-β2GP1** | 5 (7.4) | 1 (9.1) | 1 (9.1) | 0.134 |
| **ACL** | 2 (2.9) | 1 (9.1) | 0 (0) | 0.285 |
| **Elevated LA** | 0 (0) | 1 (9.1) | 0 (0) | **0.017** |
| **BNP, ng/L** | 177.9±359.03 | 393.2±357.3 | 14.7±7.2 | **0.039** |
| **NT-proBNP, pg/ml** | 1348.7±2249.1 | 1257.5±1219.9 | 113.3±131.9 | **0.033** |
| **WHO function class III-IV, NO. (%)** | 11 (16.2) | 2 (18.2) | 0 (0) | 0.392 |
| **Hemodynamics** |  |  |  |  |
| **mABP, mmHg** | 91±9 | 97±12 | 90±7 | 0.183 |
| **mPAP, mmHg** | 43±9 | 48±13 | 27±3 | **<0.001** |
| **PAWP, mmHg** | 10±3 | 18±3 | 11±2 | **<0.001** |
| **RAP, mmHg** | 8±3 | 11±4 | 7±2 | **0.003** |
| **PVR, WU** | 7.2±3.9 | 7.1±5.2 | 2.3±0.4 | **<0.001** |
| **CO, L/min** | 5.2±1.3 | 4.9±1.3 | 6.6±0.8 | **0.002** |
| **CI, L/min×m^2^** | 3.3±0.8 | 3.2±0.6 | 3.9±0.4 | **0.040** |
| **Echocardiography** |  |  |  |  |
| **IVC diameter, mm** | 14.6±3.1 | 14.0±2.7 | 13.2±1.6 | 0.558 |
| **RV diameter, mm** | 27.2±6.7 | 29.2±5.8 | 21.6±4.9 | **0.026** |
| **RV/LVEDD ratio** | 0.67±0.24 | 0.61±0.15 | 0.47±0.13 | **0.025** |
| **LVEF %** | 69.2±5.8 | 62.5±12.8 | 68.5±6.2 | 0.536 |
| **TAPSE, m0.7m** | 17.6±3.7 | 18.6±2.6 | 19.5±0.7 | 0.667 |
| **Moderate to severe mitral valve dysfunction, No. (%)** | 9 (17.0) | 4 (66.7) | 1 (11.1) | **0.049** |
| **Pericardial effusion, No. (%)** | 25 (45.5) | 2 (33.3) | 1 (111.1) | 0.138 |

WHO, World Health Organization; PH, pulmonary hypertension; IgG, Immunoglobulin G; hsCRP, hypersensitive C-reaction protein; ESR, erythrocyte dissemination rate; ANA, anti-nuclear antibodies; anti-dsDNA, anti-double-stranded DNA; anti-Sm, anti-Smith; ACL, anticardiolipin; Anti-β2GP1, anti-beta2 glycoprotein 1; anti‐RNP, antiribonucleoprotein; BNP, brain natriuretic peptide; NT-proBNP, N-terminal brain natriuretic peptide; mABP, mean arterial blood pressure; mPAP, mean pulmonary pressure; PAWP, pulmonary arterial wedge pressure; RAP, right atrium pressure; CO, cardiac output; CI, cardiac index; PVR, pulmonary arterial resistance; IVC, inferior vena cava; RV, right ventricle; LVEDD, left ventricular end-diastolic diameter; LVEF, left ventricular ejection fraction; TAPSE, Tricuspid annular plane systolic excursion.

**Table S3—Baseline and disease characteristics for IpcPH and CpcPH**

|  | **IpcPH**  **n=7** | **CpcPH**  **n=26** | **p value** |
| --- | --- | --- | --- |
| **Age, years** | 40.4±15.5 | 34.4±11.1 | 0.330 |
| **Disease duration since onset of CTD, weeks** | 4.7±8.4 | 28.2±53.3 | 0.813 |
| **BMI, kg/m2** | 20.4±1.9 | 22.4±5.5 | 0.095 |
| **BNP, ng/L** | 171.4±153.3 | 242.2±375.7 | 0.721 |
| **NT-proBNP, pg/ml** | 827.5±982.7 | 1410.6±2053.3 | 0.183 |
| **WHO functional class III-IVs, No. (%)** | 1 (14.3) | 4 (15.4) | 1.000 |
| **Hemodynamics** |  |  |  |
| **mABP, mmHg** | 88±11 | 91±14 | 0.936 |
| **mPAP, mmHg** | 38±7 | 52±9 | 0.177 |
| **PAWP, mmHg** | 21±5 | 18±3 | 0.067 |
| **RAP, mmHg** | 11±5 | 12±4 | 0.308 |
| **PVR, WU** | 2.5±0.4 | 7.9±4.3 | 0.008 |
| **CO, L/min** | 6.5±1.8 | 5.0±1.4 | 0.921 |
| **CI, L/min×m^2^** | 4.1±1.3 | 3.2±0.9 | 0.811 |
| **Echocardiography** |  |  |  |
| **IVC diameter, mm** | 15.3±1.0 | 14.4±2.3 | 0.077 |
| **RV diameter, mm** | 23.2±5.0 | 29.7±5.5 | 0.564 |
| **RV/LVEDD ratio** | 0.53±0.15 | 0.69±0.14 | 0.883 |
| **LVEF (%)** | 69±5 | 63±11 | 0.215 |
| **TAPSE, mm** | 19±3 | 17±3 | 0.267 |
| **Mitral valve regurgitation, No. (%)** | 3 (42.9) | 5 (33.3) | 1.000 |
| **Pericardial effusion, No. (%)** | 2 (28.6) | 8 (36.4) | 1.000 |
| **Treatment** |  |  |  |
| **PAH-targeted therapy before RHC, No. (%)** | 4 (57.1) | 18 (69.2) |  |
| **PAH-targeted therapy after RHC, No. (%)** | 0 (0) | 20 (76.9) |  |

IpcPH, isolated post-capillary pulmonary hypertension; CpcPH, combined pre-capillary and post-capillary pulmonary hypertension; BNP, brain natriuretic peptide; NT-proBNP, N-terminal brain natriuretic peptide; mABP, mean arterial blood pressure; mPAP, mean pulmonary pressure; PAWP, pulmonary arterial wedge pressure; RAP, right atrium pressure; CO, cardiac output; PVR, pulmonary arterial resistance; IVC, inferior vena cava; RV, right ventricle; LVEDD, left ventricular end-diastolic diameter; LVEF, left ventricular ejection fraction; PAH, pulmonary arterial hypertension; RHC, right heart catheterization.

**Table S4—Baseline and disease characteristics for CTD-PH patients with and without ILD**

|  | | | **With ILD**  **n=24** | | **Without ILD**  **n=114** | **p value** |
| --- | --- | --- | --- | --- | --- | --- |
| **Age, years** | | | 47.9±15.4 | | 34.3±9.0 | <0.001 |
| **Disease duration since onset of CTD, weeks** | | | 50.4±77.1 | | 40.1±56.8 | 0.890 |
| **BMI, kg/m2** | | | 20.7±2.9 | | 21.5±3.1 | 0.216 |
| **BNP, ng/L** | | | 131.7±140.5 | | 138.1±296.8 | 0.947 |
| **NT-proBNP, pg/ml** | | | 953.0±1226.2 | | 1296.5±3007.9 | 0.344 |
| **WHO function class III-IV, No. (%)** | | | 5 (20.8) | | 18 (15.8) | 0.552 |
| **Hemodynamics** | | |  | |  |  |
| **mABP, mmHg** | | | 88±12 | | 90±10 | 0.161 |
| **mPAP, mmHg** | | | 43±11 | | 46±11 | 0.724 |
| **PAWP, mmHg** | | | 10±3 | | 11±3 | 0.098 |
| **RAP, mmHg** | | | 7±4 | | 8±3 | 0.337 |
| **PVR, WU** | | | 6.9±3.3 | | 7.7±3.9 | 0.974 |
| **CO, L/min** | | | 5.3±1.3 | | 5.1±1.2 | 0.095 |
| **CI, L/min×m^2^** | | | 3.4±0.8 | | 3.3±0.8 | 0.072 |
| **Echocardiography** |  | | |  | | |
| **IVC diameter, mm** | | | 13.2±2.5 | | 14.4±3.1 | 0.619 |
| **RV diameter, mm** | | | 25.5±7.0 | | 28.2±7.2 | 0.929 |
| **RV/LVEDD ratio** | | | 0.62±0.20 | | 0.72±0.28 | 0.355 |
| **LVEF (%)** | | | 68±6 | | 68±9 | 0.990 |
| **TAPSE, mm** | | | 17±4 | | 17±3 | 0.074 |
| **Mitral valve regurgitation, No. (%)** | | | 4 (23.5) | | 10 (11.0) | 00.197 |
| **Pericardial effusion, No. (%)** | | 7 (35.0) | | | 29 (33.3) | 1.000 |

CTD-PH, connected tissue disease associated pulmonary hypertension; ILD, interstitial lung disease; BNP, brain natriuretic peptide; NT-proBNP, N-terminal brain natriuretic peptide; mABP, mean arterial blood pressure; mPAP, mean pulmonary pressure; PAWP, pulmonary arterial wedge pressure; RAP, right atrium pressure; CO, cardiac output; PVR, pulmonary arterial resistance; IVC, inferior vena cava; RV, right ventricle; LVEDD, left ventricular end-diastolic diameter; LVEF, left ventricular ejection fraction; PAH, pulmonary arterial hypertension; RHC, right heart catheterization.
